# Excess Potassium Promotes Autophagy to Maintain the Immunosuppressive Capacity of Myeloid-Derived Suppressor Cells Independent of Arginase 1

**DOI:** 10.3390/cells13201736

**Published:** 2024-10-19

**Authors:** Ramesh Thylur Puttalingaiah, Matthew J. Dean, Liqin Zheng, Phaethon Philbrook, Dorota Wyczechowska, Timothy Kayes, Luis Del Valle, Denise Danos, Maria Dulfary Sanchez-Pino

**Affiliations:** 1 Stanley S. Scott Cancer Center, Louisiana State University Health Sciences Center, New Orleans, LA 70112, USA; mdean3@lsuhsc.edu (M.J.D.); lzheng@lsuhsc.edu (L.Z.); pphilb@lsuhsc.edu (P.P.); dwycze@lsuhsc.edu (D.W.); tkayes@lsuhsc.edu (T.K.); ldelva@lsuhsc.edu (L.D.V.); ddanos@lsuhsc.edu (D.D.); 2 Department of Genetics, Louisiana State University Health Sciences Center, New Orleans, LA 70112, USA; 3 Department of Interdisciplinary Oncology, Louisiana State University Health Sciences Center, New Orleans, LA 70112, USA; 4 School of Public Health, Louisiana State University Health Sciences Center, New Orleans, LA 70112, USA

**Keywords:** MDSCs, autophagy, potassium, K^+^ ions, immunosuppression, arginase I

## Abstract

Potassium ions (K^+^) are critical electrolytes that regulate multiple functions in immune cells. Recent studies have shown that the elevated concentration of extracellular potassium in the tumor interstitial fluid limits T cell effector function and suppresses the anti-tumor capacity of tumor-associated macrophages (TAMs). The effect of excess potassium on the biology of myeloid-derived suppressor cells (MDSCs), another important immune cell component of the tumor microenvironment (TME), is unknown. Here, we present data showing that increased concentrations of potassium chloride (KCl), as the source of K^+^ ions, facilitate autophagy by increasing the expression of the autophagosome marker LC3β. Simultaneously, excess potassium ions significantly decrease the expression of arginase I (Arg I) and inducible nitric oxide synthase (iNOS) without reducing the ability of MDSCs to suppress T cell proliferation. Further investigation reveals that excess K^+^ ions decrease the expression of the transcription factor C/EBP-β and alter the expression of phosphorylated kinases. While excess K^+^ ions downregulated the expression levels of phospho-AMPKα (pAMPKα), it increased the levels of pAKT and pERK. Additionally, potassium increased mitochondrial respiration as measured by the oxygen consumption rate (OCR). Interestingly, all these alterations induced by K^+^ ions were abolished by the autophagy inhibitor 3-methyladenine (3-MA). Our results suggest that hyperosmotic stress caused by excess K^+^ ions regulate the mitochondrial respiration and signaling pathways in MDSCs to trigger the process of autophagy to support MDSCs’ immunosuppressive function by mechanisms independent of Arg I and iNOS. Overall, our in vitro and ex vivo findings offer valuable insights into the adaptations of MDSCs within the K^+^ ion-rich TME, which has important implications for MDSCs-targeted therapies.

## 1. Introduction

Potassium ions are the most abundant in intracellular fluid and, as such, play a vital role in cellular functions. The intracellular concentration of K^+^ ions is between 145 and 150 mM, while the extracellular concentration is lower, between 3.5 and 5.2 mM [1,2,3]. However, in solid tumors, necrotic cells release their intracellular content into the extracellular space, where K^+^ concentrations exceed 40 mM [2]. K^+^ ion signaling has been involved with molecular mechanisms that regulate malignant cell proliferation, tumor invasion, migration, and apoptosis [4]. In addition to the impact on cancer cells, K^+^ ions affect immune cells in the tumor microenvironment (TME), playing a key role in the complex crosstalk between cancer and immuno-response [5]. Recent studies have shown that K^+^ ions in the TME diminish the effector functions of T cells [3] while enhancing their stemness properties [2]. Additionally, intratumoral K^+^ ions abrogate the anti-tumor capacity of tumor-associated macrophages (TAMs), and exogenous treatment with K^+^ ions promotes the polarization of bone marrow-derived macrophages (BMDMs) toward pro-tumor M2-like TAMs [6]. These prior studies suggest that immune responses against cancer cells are influenced by K^+^ ion signaling. Thus, enhancing local anti-tumor immunity may be possible by targeting the effects of K^+^ ions on immune cells. To our knowledge, there are no studies on the impact of K^+^ ions disturbance on the biology and function of MDSCs. Instead, other studies have revealed that an excess concentration of sodium ions (Na^+^) decreases the number of tumor-infiltrating MDSCs, reduces the mRNA levels of Arg I, and blocks murine MDSC immunosuppressive functions in vitro [7,8]. Together, these findings validate our premise that excess K^+^ ions also impact the biology of MDSCs.

MDSCs are a heterogeneous immature myeloid cell subpopulation that includes monocytic (M-MDSCs) and granulocytic (G-MDSCs) subsets [9,10]. The ability of MDSCs to suppress T cell proliferation and activity [11,12] contributes to the escape of tumors from the protective immune response. MDSCs exert their T cell immunosuppression through diverse mechanisms including, but not limited, to the depletion of arginine via Arg I and iNOS [13,14]. Therefore, targeting MDSCs has been considered an effective strategy to weaken the immunosuppressive TME and enhance the efficacy of cancer immunotherapy [15,16]. Consequently, a better understanding of the biological adaptations of MDSCs within the hostile TME, in part due to the excess K^+^ ions, will provide insights into developing MDSCs-targeted therapies. Here, we mimicked the intratumoral K^+^ ions alterations in in vitro and ex vivo models to examine the impact of elevated levels of K^+^ ions on the metabolism, signaling, and function of MDSCs.

## 2. Materials and Methods

### 2.1. Mice and Tumor Model

C57BL/6J mice were purchased from Envigo (St. Louis, MO, USA) or bred in-house. An MC38 colon adenocarcinoma cell line from Kerafast (Boston, MA, USA) was cultured in RPMI-1640 media from Gibco Life Technologies (Grand Island, NY, USA), supplemented with 10% heat-inactivated fetal bovine serum (FBS) from GeminiBio (West Sacramento, CA, USA), and 25 mmol/L HEPES, 3 mmol/L L-glutamine, 100 U/mL penicillin G, and 100 μg/mL streptomycin (complete media) were all purchased from Gibco Life Technologies. MC38 cells (1.5 × 10^5^) were subcutaneously implanted into the left flank of the mice. Tumor volume was measured using calipers, and tumor volume was calculated using the formula [(small diameter)^2^ × (large diameter) × 0.5]. When the tumors reached around 300–600 mm^3^, the mice were euthanized, and the tumors were excised and processed. All experiments were approved under the Louisiana State University Health Science Center (LSUHSC)-IACUC protocol # 3872.

### 2.2. Generation of MC38-Derived Tumor Explant Supernatant (TES)

TES was prepared from excised non-ulcerated MC38 tumors ~1.5 cm in diameter or ~600 mm^3^ in tumor volume as described previously [17,18,19]. Briefly, approximately 1 g of the tumor was washed with 70% alcohol followed by 1× phosphate-buffer saline (PBS; Gibco Life Technologies) and cut into small pieces (<3–5 mm in diameter). Tumor pieces were cultured in 10 mL of RPMI media containing 100 U/mL penicillin and 100 µg/mL streptomycin, without FBS, at 37 °C with 5% CO_2_ for 18 h. After incubation, the tissue-free supernatant was collected by filtering through a 70 μm strainer (MilliporeSigma; Milwaukee, WI, USA), followed by centrifugation at 1800 rpm for 5 min. The collected supernatant was centrifuged at 13,000 rpm for 15 min at 4 °C, followed by filtration through a 0.22 μm filter (Corning Life Sciences, Tarboro, NC, USA). The TES was stored at −80 °C until it was used for in vitro experiments at indicated dilutions in complete RPMI as described below.

### 2.3. Generation and In Vitro Treatment of Mouse Bone Marrow-Induced MDSCs (mBM-MDSCs) with Cytokines or TES

mBM-MDSCs were induced using bone marrow cells in the presence of a recombinant cytokine cocktail (cytokine-induced MDSCs) or TES (TES-MDSCs) as follows. Bone marrow cells were harvested from the femur and tibia of C57BL/6 mice and resuspended in complete culture media, which contained 5 mM KCl according to the RPMI formulation (used as a control baseline of potassium) supplemented with 50 μM β-mercaptoethanol (β-Me; MP Biomedicals, LLC; Solon, OH, USA). Cytokine-induced MDSCs were propagated by culturing the bone marrow cells with 20 ng/mL each of murine recombinant Granulocyte Colony Stimulating Factor (G-CSF), Granulocyte-Macrophage Colony-Stimulating Factor (GM-CSF), and Interleukin 6 (IL-6; all were from GeminiBio) for 4 days. TES-MDSCs were induced following the model reported previously [18,19]. Briefly, bone marrow cells were cultured in complete media supplemented with β-Me (50 μM), recombinant GM-CSF (20 ng/mL), and 20%, 30%, or 40% (*v*/*v*) TES for 2 days. At 48 h of culture, fresh media containing the same amount of TES, GM-CSF, and the specific treatment was added to the tissue culture and incubated for another 48 h. To determine the effect of excess K^+^ ions, potassium chloride (KCl; MilliporeSigma) at 20, 30, or 40 mM was added to the tissue culture during the MDSCs differentiation. For autophagy inhibition, 0.5 mM 3-methyladenine (3-MA; Santa Cruz Biotechnology; Dallas, TX, USA) was added to the bone marrow cell culture, and after 1 h of incubation, the cells were treated with 40 mM KCl and cultured for 4 days. After 96 h, cytokine-induced MDSCs and TES-MDSCs were collected and processed for further experiments.

### 2.4. Hematoxylin and Eosin (H&E) and Immunofluorescence (IF)

Cytokine-induced MDSCs (0.15 × 10^6^) were seeded on a 0.001% Poly-L-Lysine (Millipore-Sigma, Milwaukee, WI, USA)-coated microscope slide (ThermoFisher Scientific, Carlsbad, CA, USA) using a 3-well Cell Concentrator (Avantor-VWR, Radnor, PA, USA) in 100 μL of its own supernatant from 4 days of culturing for 1 h at 37 °C in a CO_2_ incubator. After spinning down at 1000 rpm for 1 min, the cells were fixed in cold 100% methanol for 10 min, rinsed with 1× PBS, and stained with H&E or labeled for immunofluorescence (IF). H&E staining was conducted according to standard protocols. Autophagosomes were detected by IF as previously described [20]. In detail, fixed cells on the microscope slide, including a positive control of treated MDSCs with 1 μg/mL of tunicamycin (MilliporeSigma) for the last 24 h of culture, were incubated in a blocking buffer (5% goat serum [ThermoFisher Scientific] in 0.25% Triton X-100 [MilliporeSigma] diluted in 1× PBS) for 1 h at room temperature (RT). Then, the cells were incubated with a primary rabbit monoclonal anti-LC3β antibody (Cell Signaling Technology, Danvers, MA, USA) at a 1:2000 dilution in an antibody dilution buffer (1% goat serum/0.25% Triton X-100 PBS) overnight at 4 °C in a humidified chamber. After thoroughly rinsing in 1× PBS, the secondary anti-rabbit antibody tagged with Alexa-Fluor 488 (ThermoFisher Scientific) at a 1:200 dilution in an antibody dilution buffer was added and incubated for 1 h at RT in a humidified chamber protected from light. Finally, the cells were rinsed in 1× PBS, and the slide was mounted with the aqueous-based mounting media containing DAPI (ProLong Gold Antifade, Invitrogen from ThermoFisher Scientific) and imaged using an Olympus Fluoview FV1000 confocal microscope (Olympus, Center Valley, PA, USA). The number of LC3-positive puncta per cell was determined using the Analyze Particles tool in ImageJ in 3 different pictures per condition.

### 2.5. Characterization of MDSCs by Flow Cytometry

The following fluorochrome-conjugated anti-mouse antibodies were used to characterize MDSCs subtypes: BV421-anti-Gr-1^+^ (RB6–8C5), APC-anti-Ly6G (1A8), FITC-anti-Ly6C (AL-21), BV711-anti-F4/80 (T45-2342), and PE-anti-CD45 (MIH5), and purified anti-CD16/32 (2.4G2) was used for blocking Fc receptors. The antibodies were purchased from BD biosciences (Becton Dickinson; Sparks, MD, USA), and FITC-anti-CD45 (30-F11) and APC-eFluor780-anti-CD11b (M1/70) from Invitrogen (ThermoFisher Scientific). The stained cells were examined by flow cytometry using 8-color Gallios (Beckman Coulter; Brea, CA, USA). The data were analyzed using Kaluza software (Beckman Coulter, Version 2.1).

### 2.6. Quantification of Potassium

Complete media (RPMI), 100% TES, 20% TES diluted in complete media, and 20% TES supplemented with 40 mM KCl were used to quantify the concentration of K^+^ ions using a Potassium Assay Kit (Abcam; Waltham, MA, USA) following the manufacturer’s instructions.

### 2.7. Isolation of Tumor-Infiltrating MDSCs (T-MDSCs) and Ex Vivo Treatment

Dissected subcutaneous MC38 tumors were digested with 100 μg/mL of DNAse I and 0.5 Wünsch U/mL of Liberase (Roche^®^ Life Science Products from MilliporeSigma) at 37 °C with rotation at 14 rpm for 30 min to obtain a single-cell suspension. Gr1^+^ cells (T-MDSCs) were isolated using the EasySep Mouse Gr-1 positive selection kit (STEMCELL Technologies; Vancouver, BC, Canada). Purity (>75%) was assessed by flow cytometry. Isolated Gr-1^+^ cells were cultured with complete RPMI supplemented with GM-CSF (20 ng/mL) in the presence or absence of 40 mM KCl. After 16 h of incubation, the T-MDSCs were collected and processed for further experiments.

### 2.8. Cytotoxicity Assays

The effect of excess potassium on cell viability was performed by quantifying healthy, apoptotic, and necrotic cells by assessing phosphatidylserine exposure and loss of membrane integrity with vital dyes through end-point and real-time assays. These assays include the selective staining of dead cells with trypan blue and examination using an automated cell counter, staining with Annexin V and propidium iodide solution followed by flow cytometry analysis and the real-time readout of cell toxicity in a high-throughput screening format. For the trypan blue exclusion end-point assay, MDSCs were harvested after in vitro culture, washed, and resuspended in 1 mL of 1× PBS. The cells were mixed with a 0.4% trypan blue stock solution (Invitrogen from ThermoFisher Scientific) at a 1:10 dilution and loaded into a hemacytometer, followed by the quantification of viable and dead cells using a BD automated cell counter (ThermoFisher Scientific).

For the annexin V (AnnV) and propidium iodide (PI) end-point assay, the cells were stained with FITC- AnnV and PI according to the manufacturer’s instructions (FITC Annexin V apoptosis detection kit (Becton Dickinson)). The determination of dead (AnnV^+^PI^+^, AnnV^+^PI^−^, AnnV^−^PI^+^) and alive cells (AnnV^−^PI^−^) was performed using a Gallios flow cytometer. Dead cells induced by exposure to high temperatures (3 min at 70 °C) and mixed with healthy cells were used as a positive control for flow cytometry acquisition.

To monitor toxicity during the in vitro culture, a mix-and-read, real-time quantification of cell death method was performed using the non-permeable Incucyte Cytotox Red Dye (Sartorious, Ann Arbor, MI, USA) according to the manufacturer’s instruction. Briefly, 0.1 × 10^6^ bone marrow cells in 100 μL complete media containing recombinant cytokines, 20 mM, or 40 mM KCl, in the presence with 250 nM Incucyte Cytotox Red Dye, were seeded into a 96-well plate. The cells were incubated in the Incucyte Live-Cell Analysis System (Sartorious), and images were captured every 12 h (20×; 4 images per well) to record the cells becoming unhealthy. At the end of the 4 days of readouts, the efficacy of the Cytotox Red Dye was assessed by adding a detergent (1:200 dilution of Lysis Solution (Promega, Madison, WI, USA)) into a well to enable the staining of the entire cell population. As a background control, cells without Cytotox Red Dye were included. The analysis involved measuring cell death by counting red objects in combination with the Incucyte confluence metric.

### 2.9. Isolation of T Cells

CD3^+^ cells were isolated from the spleen and lymph nodes of C57BL/6J using a mouse T cell negative isolation kit (Invitrogen from ThermoFisher Scientific) according to the manufacturer’s instructions. Purity exceeded > 90% by FACS analysis (Gallios).

### 2.10. Assessment of MDSCs Suppressive Function on T Cell Proliferation

Mouse CD3^+^ T cells were labeled with 5 nM CellTrace^TM^ violet dye according to the manufacturer’s instructions (Invitrogen from ThermoFisher Scientific). Cytokine-induced MDSCs, TES-MDSCs, or T-MDSCs, cultured with or without K^+^ ions, were washed and cocultured with CellTrace^TM^ violet dye-labeled T cells at different ratios of MDSCs/T cells. The activation of T cells was performed using Dynabeads^TM^ Mouse T-Activator CD3/CD28 kit (Gibco from ThermoFisher Scientific) at a beads/T cell ratio of 0.5:1 in the presence of mouse recombinant IL-2 (50 U/mL; R&D Systems; Minneapolis, MN, USA) according to the manufacturer’s instructions. After 72 h of coculture, CellTrace^TM^ dilution on T cell proliferation was measured using a Gallios flow cytometer (Beckman Coulter; Brea, CA, USA) and analyzed with Kaluza software (Beckman Coulter, 2.1 version).

### 2.11. Arginase Activity Assay

Cell lysates from mBM-MDSCs were evaluated for arginase activity by measuring the production of L-ornithine as previously described [21,22]. Briefly, cell lysates (5 μg) or L-ornithine standards (from 1 to 250 nM) were incubated with 25 μL of 10 mM MnCl_2_ at 55 °C for 20 min to activate the enzymatic activity of arginase. Then, the samples were incubated at 37 °C for 20 min in the presence of 150 μL of 100 mM carbonate buffer (MilliporeSigma) and 50 μL of 100 mM L-arginine. The reaction was halted by adding 750 μL of glacial acetic acid. The hydrolysis reaction from L-arginine to L-ornithine by arginase was measured by adding 250 μL of ninhydrin solution, followed by incubation for 1 h at 95–100 °C. The colorimetric reaction was quantified by spectrophotometry at 570 nm (Bio-Rad benchmark microplate reader; Hercules, CA, USA). All these chemicals were purchased from MilliporeSigma. The level of arginase activity is expressed as nM of ornithine by 5 μg of protein produced in 20 min of enzymatic reaction.

### 2.12. Measurement of Nitrate Concentration

Nitric oxide production was indirectly assessed by quantifying nitrite levels in cell lysates from mBM-MDSCs using the colorimetric assay with Griess Reagent. Briefly, cell lysates (10 μg) or sodium nitrite standards (from 1 to 100 μM) were incubated with Griess Reagent (Invitrogen from ThermoFisher Scientific) for 30 min at RT, and the concentration of nitrite was quantified spectrophotometrically at 548 nm (Bio-Rad benchmark microplate reader).

### 2.13. Western Blotting

Cell lysates were obtained with M-PER™, Mammalian Protein Extraction Reagent (ThermoFisher Scientific). For the extraction of cytoplasmic and nuclear proteins, the cells were washed with ice-cold PBS and lysed with cytoplasmic extract (CE) buffer (10 mM HEPES pH 7.9, 10 mM KCl, 0.1 mM EDTA, 0.3% NP-40) and 1× protease inhibitors of the Halt Protease Inhibitor Cocktail (ThermoFisher Scientific). After the separation of the CE by centrifugation (3000 rpm, 5 min, 4 °C), the pellet was lysed with nuclear extract (NE) buffer (20 mM HEPES pH 7.9, 0.4 mM NaCl, 0.1 mM EDTA, 25% glycerol and 1× protease inhibitors). The protein content in the cell extracts was measured using the Bicinchoninic acid assay (BCA) method (Pierce^TM^ Rapid Gold BCA Protein Assay Kit from ThermoFisher Scientific) according to the manufacturer’s instructions. Aliquots of lysates containing 40 μg of protein/gel lane were electrophoresed on 1.5 mm-thick 4–12% Tris-Glycine gels (ThermoFisher Scientific) under reducing conditions. The proteins were electroblotted onto PVDF membranes and immunoblotted with antibodies against pAMPKα (Thr172; clone 40H9), AMPKα (clone D5A2), pAkt (Ser473; clone D9E), Akt (clone 40D4), pERK (Thr202/Tyr204; clone D13.14.4E), ERK (clone L34F12), LC3β (clone D11), and HDAC1 (clone D5C6U) (Cell Signaling Technology), C/EBP-β (clone H-7; Santa Cruz Biotechnology), Arg I (clone 19; BD Biosciences), iNOS (clone 54; BD Biosciences), and β-actin (clone AC-74; MilliporeSigma). The membranes were developed using the Kwik Quant Western blot detection kit, and images were taken using a Kwik Quant Imager (Kindle Biosciences, LLC, Greenwich, CT, USA), followed by analysis with NIH ImageJ software (Version 154).

### 2.14. RNA Isolation and Reverse Transcriptase Quantitative Polymerase Chain Reaction (RT-qPCR)

The control and potassium-treated mBM-MDSCs were cultured for 96 h and then lysed with RLT containing 2-mercaptoethanol (MilliporeSigma) and passed through a QI shredder (Qiagen; Germantown MD, USA) column before extraction using the RNeasy mini kit (Qiagen), following the manufacturer’s instructions, with an on-column DNase digestion step (Qiagen). RNA (1 μg) was transcribed into cDNA using a Verso cDNA synthesis kit (ThermoFisher Scientific), and quantitative PCR was conducted in technical triplicate using the QuantStudio 12K flex system (Applied Biosystems by Life Technologies; Waltham, MA, USA). The primers (Integrated DNA Technologies, Inc.; Coralville, IA, USA) used for cDNA amplification were as follows: Arg I, forward 5′-CGG GAG GGT AAC CAT AAG GC-3′ and reverse 5′-GTC TGC TTT GCT GTG ATG GC-3′; iNOS forward 5′-CCT GCT TTG TGC GAA GTG TC-3′ and reverse 5′-CCC AAA CAC CAA GCT CAT GC-3′; and β-actin forward 5′-GAT CAA GAT CAT TGC TCC TCC TGA-3 and reverse 5′-CAG CTC AGT AAC AGT CCG CC-3′. After the reaction, the relative fold changes in mRNA expression levels were calculated using ΔΔCt (ddCt), normalized to the housekeeping gene β-actin.

### 2.15. Measurement of Oxygen Consumption Rate and Extracellular Acidification Rate

Oxygen consumption rate (OCR) and extracellular acidification rate (ECAR) were measured with a Seahorse XF96 Analyzer (Agilent Technologies, Santa Clara, CA, USA). In total, 1.3 × 10^5^ mBM-MDSCs were seeded per well in a Cell-Tak (BD Biosciences)-coated Seahorse 96-well plate with the Seahorse XF RPMI Assay Medium (Agilent Technologies) supplemented with 10 mM glucose, 2 mM glutamine, and 1 mM sodium pyruvate (Agilent Technologies). OCR measurements were recorded at baseline, followed by sequential additions of 1.5 μM oligomycin, 1 μM carbonyl cyanide p-trifluoromethoxyphenylhydrazone (FCCP), and 0.5 μM rotenone/antimycin A (Seahorse XF Cell Mito Stress Test Kit, Agilent Technologies). The ECAR of mBM-MDSCs was measured in XF RPMI media containing 2 mM L-glutamine under basal conditions and in the presence of 10 mM L-glucose, 1 μM oligomycin, and 50 mM 2-DG (Seahorse XFp Glycolysis Stress Test Kit, Agilent Technologies). The OCR and ECAR results were normalized to cell number (1000 cells) and expressed as pmol/min/1000 cells and mpH/min/1000 cells, respectively. The nucleic acid staining was performed by including 5 μM Hoechst (ThermoFisher Scientific) into the rotenone/antimycin A injection. The Mito Stress Test and Glycolysis Stress Test data were analyzed using the Seahorse Report generator (Agilent Technologies).

### 2.16. Measurement of Cytokines and Chemokines by Multiplex

Cytokines and chemokines were assessed from the control and treated mBM-MDSCs culture supernatants using the Cytokine and Chemokine Convenience 36-Plex Mouse ProcartaPlex™ Panel 1A (Invitrogen from ThermoFisher Scientific) as per the manufacturer’s instructions. Cytokine/Chemokine detection and quantification were performed with a MAGPIX from Luminex (Austin, TX, USA).

### 2.17. Glucose Uptake Assay 

Control and KCl-treated mBM-MDSCs (1 × 10^6^) were washed with 1× PBS and subsequently incubated with 50 µM fluorescent glucose analog 2-(N-[7-nitrobenz-2-oxa-1,3-diazol-4-yl] amino)-2-deoxyglucose (2NBDG; Invitrogen from ThermoFisher Scientific) in 1× PBS for 2 h at 37 °C. After incubation, the cells were washed with 1× PBS and then stained with fluorochrome-labeled antibodies for MDSCs surface markers for 20 min at RT. The mean fluorescence intensity (MFI) of 2-NBDG was measured by Gallios’ flow cytometer.

### 2.18. Statistical Analysis

All data are presented as the mean ± standard deviation (SD). Data visualization and statistical analysis were performed using Prism 6, Version 6.07 (GraphPad Software Inc., La Jolla, CA, USA). Differences between the two groups were evaluated using unpaired *t*-tests. Differences between more than two groups were compared using two-way ANOVA with Tukey’s multiple comparisons test. Details of statistical analysis, including sample numbers (*n*), are included in the respective figure legends. The difference was considered statistically significant when *p* < 0.05.

## 3. Results

### 3.1. Excess Potassium Does Not Affect the Viability of mBM-MDSCs or the Expansion of MDSCs Subsets, nor Does It Significantly Affect the Autocrine Production of Cytokines and Chemokines

**Figure 1:**
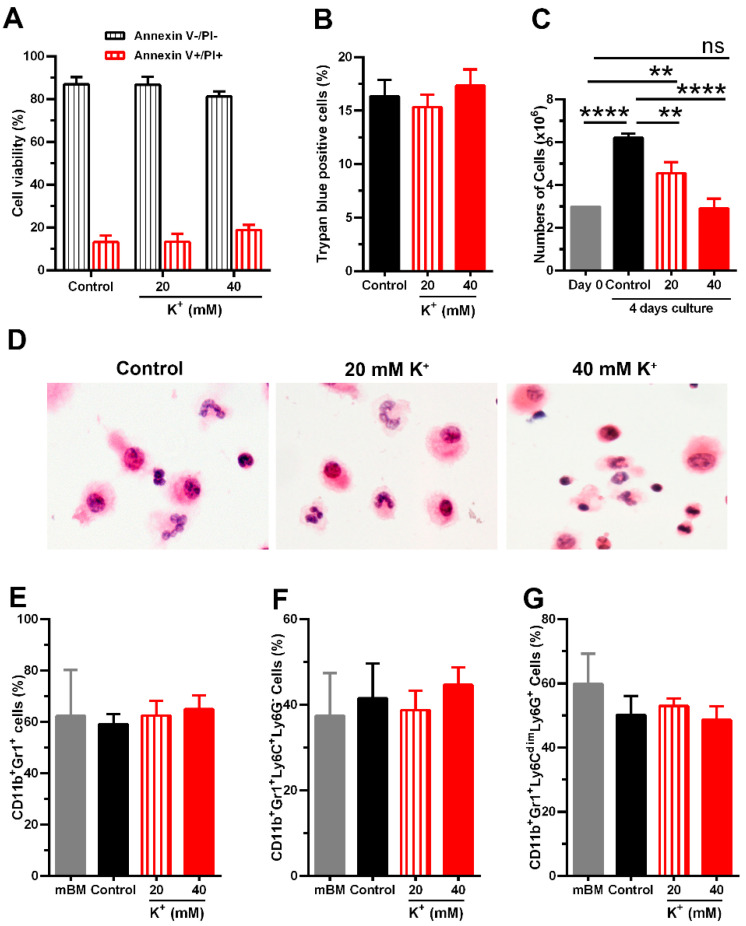
Effect of excess K^+^ ions on the viability, morphology, and differentiation of MDSCs from bone marrow (mBM-MDSCs). (**A**) The percentage of dead (Annexin V+/PI+) or viable (Annexin V−/PI−) cytokine-induced mBM-MDSCs was measured by the Annexin V/Propidium iodide (PI) staining method. (**B**) The percentage of cell death measured by trypan blue exclusion was assessed by an automated cell counter. (**C**) Comparison of the number of cells at the beginning of culture (Day 0) and at the end of culture (Day 4) assessed by the trypan blue exclusion method. (**D**) Morphologic features of mBM-MDSCs by H&E staining after exposure to indicated concentrations of KCl for 96 h (represented as potassium, K^+^ ions) and a representative image taken at 600× magnification. (**E**) Percentages of CD11b^+^Gr-1^+^ (total-MDSC), (**F**) CD11b^+^Gr1^+^Ly6C^+^Ly6G^−^ (M-MDSC), and (**G**) CD11b^+^Gr1^+^Ly6C^dim^Ly6G^+^ (G-MDSC) subsets were calculated from flow cytometry data after treatment with the indicated concentrations of KCl. Control indicates cytokine-induced mBM-MDSCs cultured in complete media that contains 5 mM KCl. mBM indicates fresh mouse bone marrow cells as primary cells used to induce in vitro mBM-MDSCs before any treatment with KCl. Data represents the mean ± SD of three independent experiments. ** *p* < 0.01, **** *p* < 0.0001 (one-way ANOVA with Tukey’s multiple comparisons test).

We first evaluated the cytotoxic effect of K^+^ ions on cytokine-induced MDSCs after four days of culture. The results show that treatment with 40 mM, but not 20 mM KCl, resulted in ~18% cell death, determined by Annexin V^+^/PI^+^, compared to the control cells (~12%; Figure 1A and Supplementary Figure S1A). Like the AnnV/PI results, the trypan blue exclusion method also showed an insignificant number of dead cells treated with 40 mM KCl compared with non-treated cells (Figure 1B). Interestingly, we noticed that the total number of cells obtained after the four days of mBM-MDSCs induction was significantly reduced by the 40 mM KCl treatment (Figure 1C), suggesting that excess potassium restricted the ability of cells to proliferate during the culture. As shown in Figure 1C, while control cells doubled the number of cells, the number of cells increased ~1.5 times in the treatment with 20 mM KCl, but did not increase in the presence of 40 mM KCl. The effect of excess K^+^ ions on both toxicity and the proliferative capacity of cells in culture was also confirmed by the real-time quantification of cells positive for Cytotox Red Dye and the percentage of confluence using the Incucyte Live-Cell Analysis System (Supplementary Figure S1B–D). Since 40 mM KCl is the level of K^+^ ions observed in the TME [2], and this did not induce toxic effects on cultured MDSCs, further experiments were performed using this concentration.

To further assess how potassium impacts the morphology of MDSCs, microscope slide preparations from these cells were stained with H&E. Microscopic evaluation showed no differences in cell morphology in treated cells with KCl (Figure 1D). However, following the standards for identifying MDSCs [23,24,25], we also measured surface expression markers to identify MDSCs. The impact of excess K^+^ ions on MDSC subtypes was quantified using flow cytometry. K^+^ ions did not influence either the percentage of total MDSCs (CD11b^+^Gr-1^+^) or M-MDSCs (CD11b^+^Gr1^+^Ly6C^+^Ly6G^−^) or G-MDSCs (CD11b^+^Gr1^+^Ly6C^dim^Ly6G^+^) subsets obtained after culture (Figure 1E–G and Supplementary Figure S2). Together, these observations suggest that the hyperosmotic stress caused by increased K^+^ ions do not cause cell toxicity or alter the induction of M-MDSCs and G-MDSCs subsets, but instead regulates the machinery for cell proliferation triggered by growth factors.

**Figure 2:**
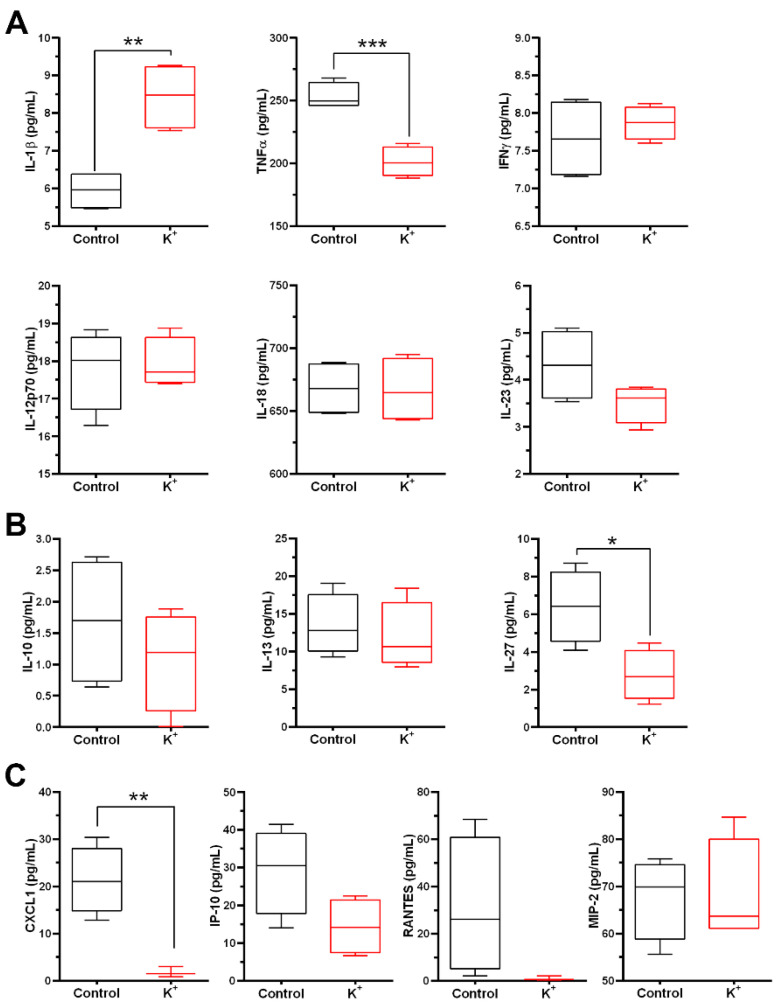
Effect of excess K^+^ ions on cytokines and chemokines produced by mBM-MDSCs. The indicated pro-inflammatory (**A**) and anti-inflammatory cytokines (**B**) and chemokines (**C**) were measured using a multiplex from the cell culture supernatant of cytokine-induced MDSCs treated or not treated with KCl as K^+^. The control indicates that MDSCs were induced in complete media. The data represent the mean ± SD of at least three independent experiments. * *p* < 0.05, ** *p* < 0.01, *** *p* < 0.001 (unpaired two-tailed *t*-test analysis).

Further, by dissecting how MDSCs respond to excess K^+^ ions, we measured cytokines and chemokines produced during MDSCs expansion and activation in vitro. As shown in Figure 2A–C, the treatment with 40 mM KCl did not dramatically alter the cytokines/chemokines production by MDSCs. However, a significantly increased production of the pro-inflammatory cytokine IL-1β, and decreased TNFα compared to the control (Figure 2A), were observed. Among the anti-inflammatory cytokines and chemokines, IL-27 (Figure 2B) and CXCL1 were decreased by K^+^ ions (Figure 2C). These results indicate that although elevated levels of K^+^ ions led to changes in the production of a few cytokines and chemokines, they did not have significant effects that ultimately shifted the balance between pro- and anti-inflammatory profiles.

### 3.2. Excess Potassium Decreases the Expression of Molecules Related to Immunosuppression

**Figure 3:**
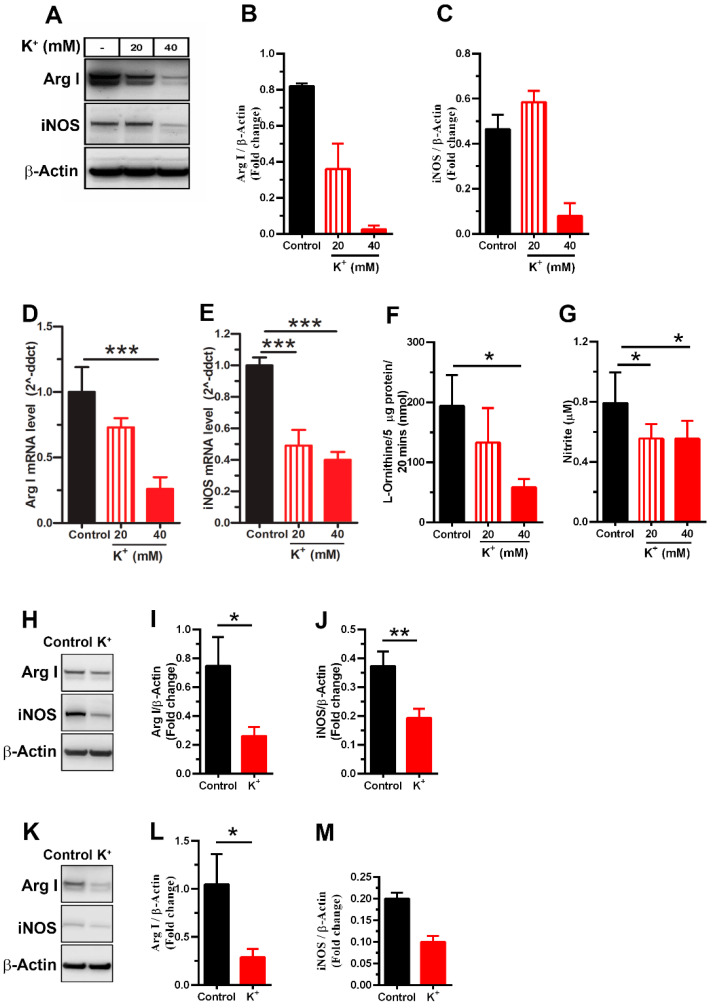
Excess potassium decreases the expression of Arg I and iNOS in mBM-MDSCs. (**A**) Representative immunoblots of Arg I and iNOS from cytokine-induced MDSCs. (**B**,**C**) Protein levels of Arg I and iNOS relative to β-actin were analyzed by densitometry (NIH ImageJ software). mRNA levels of (**D**) Arg I and (**E**) iNOS were analyzed by RT-qPCR. (**F**) Arginase activity was measured by the amount of L-ornithine produced in a biochemical in vitro assay. (**G**) Nitric oxide production was detected by measuring the concentration of nitrite in cellular lysates. The protein expression of Arg I and iNOS by Western blotting in (**H**) TES-MDSCs and (**K**) T-MDSCs with their corresponding (**I**,**J**,**L**,**M**) densitometric analysis for protein levels relative to β-actin. The control indicates MDSCs induced in complete media containing 5 mM KCl. The data shown are representative of three (**D**–**L**) or two (**A**–**C**,**M**) independent experiments. * *p* < 0.05, ** *p* < 0.01, *** *p* < 0.001 (one-way ANOVA with Tukey’s multiple comparisons test or unpaired two-tailed *t*-test analysis for experiments with *n* ≥ 3).

MDSCs inhibit T cell proliferation and activation through diverse mechanisms including, the depletion of arginine by the enzymatic activity of Arg I and iNOS [24,26,27]. We evaluated the effect of excess K^+^ ions on the expression of these molecules in cytokine-induced MDSCs. For Arg I, both mRNA and protein levels decreased in a dose-dependent manner (Figure 3A,B,D), which correlated with reduced arginase activity (Figure 3F). For iNOS, a dramatic reduction was observed with 40 mM KCl (Figure 3A,C,E). Additionally, nitrite concentration, an indirect indicator of nitric oxide production, decreased at both 20 and 40 mM KCl (Figure 3G).

We also evaluated the effect of K^+^ ions on Arg I and iNOS expression in TES-MDSCs and MC38 infiltrating Gr1^+^ cells (T-MDSCs). To prevent potassium overload and unwanted toxic effects on cells during the mBM-MDSCs induction with TES, we first measured the potassium concentration in TES and its impact on cell viability after culture. The potassium concentrations in the different media to induce MDSCs were 62.3 mM in 100% TES, 10.5 mM in 20% TES, 48 mM in 20% TES supplemented with exogenous 40 mM KCl, and 4.5 mM in complete media. The treatment of MDSCs with TES enriched with potassium did not influence the viability of cells (Supplementary Figure S3). Therefore, the 40 mM KCl in the 20% TES condition was selected as the working concentration for inducing TES-MDSCs in our experiments. Like cytokine-induced MDSCs, high concentrations of extracellular K^+^ ions decreased the expression of Arg I and iNOS in both TES-MDSCs (Figure 3H–J) and T-MDSCs (Figure 3K–M). Collectively, these results suggest that excess K^+^ ions reduce the expression of the key functional molecules Arg I and iNOS in MDSCs.

### 3.3. Potassium Alters the Expression of Intracellular Signaling Molecules in MDSCs

**Figure 4:**
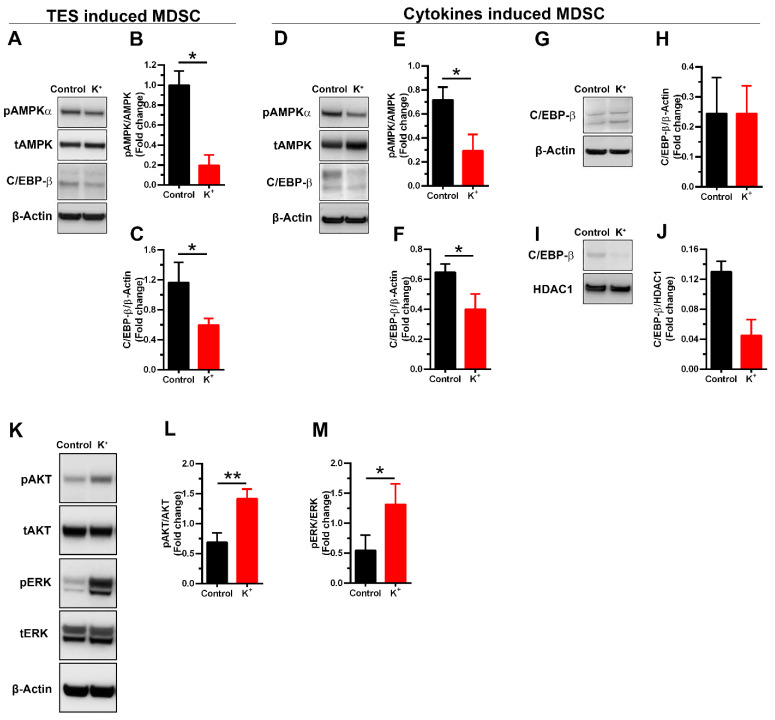
Excess K^+^ ions impair the C/EBP-β expression and phosphorylation levels of AMPK, Akt, and ERK. (**A**) Representative immunoblots of pAMPKα, total AMPK, and C/EBP-β from whole-TES-MDSCs extract. Quantifications of (**B**) pAMPKα and (**C**) C/EBP-β were determined by densitometry relative to β-actin. Cells cultured with TES and GM-CSF without KCl are considered a control. Representative immunoblots and densitometric quantification of pAMPKα, tAMPK, and C/EBP-β from (**D**–**F**) whole-cell extract, and C/EBP-β from cytosolic (**G**,**H**) and (**I**,**J**) nuclear cell extract from cytokine-induced MDSCs. (**K**) Representative immunoblots of pAkt and pERK, and their corresponding total proteins Akt and total ERK, from cytokine-induced mBM-MDSCs, treated or not treated with KCl as K^+^. The abundances of (**L**) pAkt and (**M**) pERK were determined by densitometry relative to Akt and ERK, respectively. Control indicates MDSCs induced in complete media that contain 5 mM KCl. Data represent mean ± SD of at least three independent experiments. (**G**–**J**) Data are representative of two independent experiments. * *p* < 0.05, ** *p* < 0.01 (unpaired two-tailed *t*-test analysis for experiments with *n* ≥ 3).

To elucidate the molecular mechanisms responsible for potassium impacting both the proliferation of MDSCs and the expression of Arg I and iNOS, we examined the protein expression levels of AMPK, C/EBP-β, and Akt. These signaling molecules are known to govern survival, expansion, metabolism, activation, and the polarization of myeloid precursors into immunosuppressive MDSCs via the regulation of Arg I and NOS2 expression, among others [19,28,29,30,31,32]. As shown in Figure 4, we observed a significant decrease in the expression levels of pAMPK and C/EBP-β in whole-cell extracts of TES-MDSCs (Figure 4A–C) and cytokine-induced MDSCs (Figure 4D–F). As expected, the reduced levels of C/EBP-β were primarily observed in the nuclear extract (Figure 4I,J) compared to in the cytoplasmic extract (Figure 4G,H) of cytokine-induced MDSCs. On the contrary, KCl treatment increased the level of pAkt (Figure 4K,L), a kinase that negatively regulates the activation of both C/EBP-β and AMPK [33,34].

Given that the mitogen-activated protein kinase (MAPK)/extracellular signal-regulated kinase (ERK) pathway also plays an important role in the functionality of tumor-infiltrating MDSCs [19], and the activation of ERK impairs the catalytic activity of AMPK [35], we also assessed the level of pERK and found that potassium significantly increased the level of the phosphorylated form of the kinase (Figure 4K,M), which agrees with previous reports indicating that potassium increases the ERK signaling pathways in different types of cells [36,37]. Collectively, these results suggest that the downregulation of both C/EBP-β expression and levels of pAMPK, as well as the increased phosphorylation levels of Akt and ERK pathways by excess K^+^ ions, lead to phenotypic changes in MDSCs.

### 3.4. Potassium Enhances Autophagy Pathway Activation and Mitochondrial Oxidation in mBM-MDSCs

**Figure 5:**
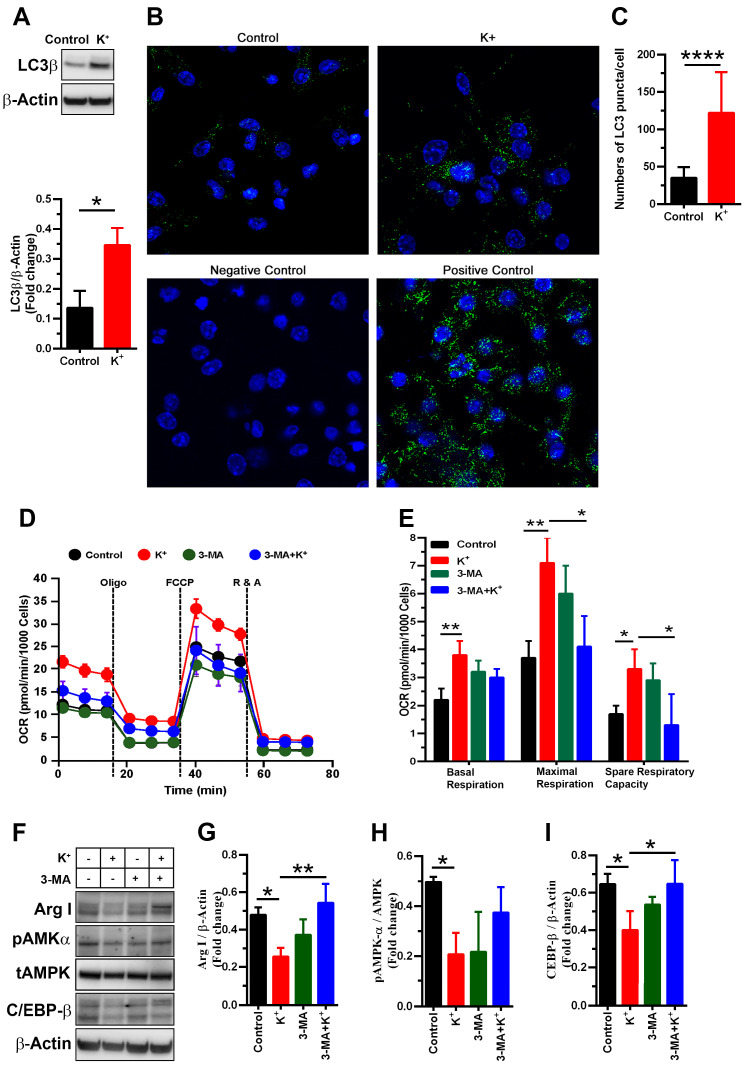
Potassium increases autophagy, and its inhibition with 3-methyladenine (3-MA) decreases mitochondrial respiration in mBM-MDSCs exposed to K^+^ ions. (**A**) Immunoblot (upper panel) and densitometric quantification (lower panel) of LC3β in cytokine-induced MDSCs treated or untreated with K^+^ ions of three independent experiments. * *p* < 0.05 (unpaired two-tailed *t*-test analysis). (**B**) Representative immunofluorescent images (600× magnification) of endogenous LC3 puncta/autophagosome formation upon the induction of autophagy in mBM-MDSCs. Two different controls are included, the negative control, establishing the background, consists of cells without the secondary FITC-labeled antibody, and the positive control consists of cells exposed to 1 μg/mL tunicamycin for 24 h. The nuclei are in blue (DAPI) and the LC3 puncta are in green. Cells in complete media (5 mM KCl) were considered the controls. (**C**) The number of LC3 puncta per cell was determined using an ImageJ quantification tool for ≥21 randomly selected cells. **** *p* < 0.0001 (unpaired two-tailed *t*-test analysis). (**D**) Representative plot of OCR measurements under basal conditions and after the addition of indicated drugs on cytokine-induced MDSCs pre-treated with or without 3-MA and 40 mM KCl. (**E**) Bioenergetic measurements were conducted by monitoring OCR values over time and under specified injections as shown in (**D**) using a Seahorse extracellular flux analyzer. The data are shown as means ± SDs of four independent experiments. * *p* < 0.01, ** *p* < 0.001 (one-way ANOVA with Tukey’s multiple comparisons test). (**F**) Immunoblot of Arg I, pAMPKα, tAMPK, and C/EBP-β from cytokine-induced MDSCs pre-treated with or without 3-MA and 40 mM KCl. (**G**–**I**) Protein expression of Arg I and C/EBP-β relative to β-actin, and pAMPKα relative to AMPK, were analyzed by densitometry (NIH ImageJ software). The data shown are representative of three independent experiments. * *p* < 0.05, ** *p* < 0.01 (one-way ANOVA with Tukey’s multiple comparisons test).

AMPK and C/EBP-β have been previously involved in regulating cellular metabolism [29,30,31,32] and autophagy [38,39,40]. Therefore, we sought to determine whether changes in the expression of C/EBP-β and levels of pAMPK and by excess K^+^ ions correlated with the expression of the autophagosome marker LC3β and cellular metabolism. As expected, the in vitro induction of mBM-MDSCs led to a time-dependent increase in LC3β expression (Supplementary Figure S4) in both the control and KCl-treated MDSCs; however, the effect of treatment with KCl in LC3β expression reached statistical significance only towards the end of the culture period (between 72 and 96 h; lower panel Figure 5A, and Supplementary Figure S4). The presence of autophagosomes was further confirmed by visualizing and quantifying cytoplasmic LC3 puncta (Figure 5B,C).

**Figure 6:**
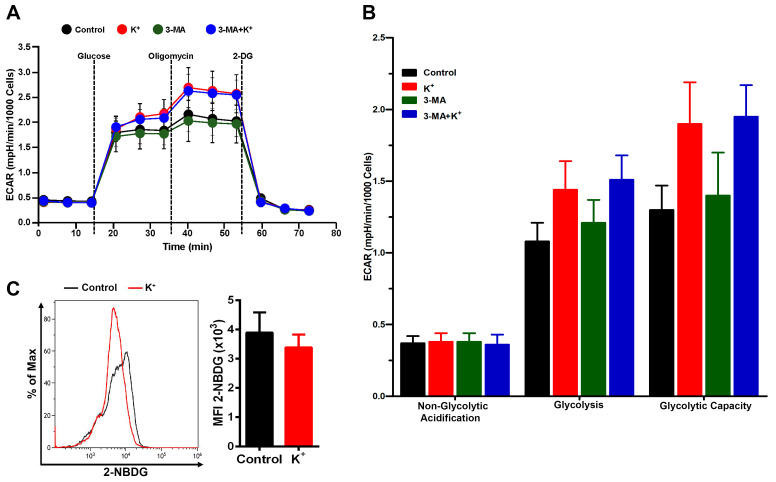
Autophagy inhibition with 3-MA does not impact glycolytic parameters of mBM-MDSCs exposed to excess K^+^ ions. (**A**) Representative plot of ECAR by Seahorse extracellular flux analyzer showing basal conditions and after the addition of the indicated drugs of cytokine-induced MDSCs pre-treated with or without 3-MA and 40 mM KCl. (**B**) Measurement of ECAR values over time and under-indicated injections as shown in (**A**). The data are shown as the means ± SDs of two independent experiments (one-way ANOVA with Tukey’s multiple comparisons test). (**C**) 2-NBDG (Glucose) uptake from control and K^+^ ion-treated cytokine-induced MDSCs were measured by flow cytometry as shown in a representative histogram, as well as the quantification from three independent experiments. The data are shown as means ± SDs of at least three independent experiments (unpaired two-tailed *t*-test analysis).

Given that autophagy is highly regulated by cellular metabolism [41], we further explored the impact of potassium on both mitochondrial respiration and the glycolytic capacity of MDSCs. As shown in Figure 5D,E, excess K^+^ ions elevated the oxygen consumption rate (OCR), indicating enhanced mitochondrial respiration. We also evaluated whether potassium-induced autophagy might be necessary to meet the increased bioenergetic demands of MDSCs under a microenvironment rich in K^+^ ions. The treatment of cells with 3-MA (0.5 mM), an autophagosome inhibitor, not only reduced the elevated levels of OCR promoted by K^+^ ions (Figure 5D,E), but also rescued the expression of Arg I (Figure 5F,G) and C/EBP-β (Figure 5F,I), and partially rescued the level of pAMPKα (Figure 5F,H) inhibited by K^+^ ions. Further, we also tested whether potassium could impair functional energy restriction [2] by assessing glycolysis and glucose uptake. Our results show that neither K^+^ ion treatment alone nor that of K^+^ ions with 3-MA had a significant impact on ECAR parameters (non-glycolytic acidification, glycolysis, and glycolytic capacity) and glucose uptake (Figure 6A–C). Together, these results suggest that excess K^+^ ions induce signaling pathways involved in preserving mitochondrial fitness to sustain prolonged autophagy in MDSCs.

### 3.5. Excess Potassium Does Not Affect T Cell Suppression Mediated by MDSCs

**Figure 7:**
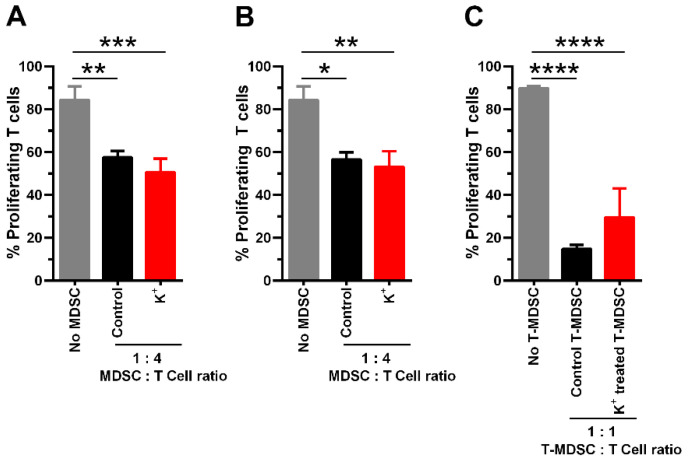
Excess K^+^ ions do not affect the ability of MDSCs to suppress T cell proliferation. The immunosuppressive function is shown by the ability of (**A**) cytokine-induced MDSCs, (**B**) TES-MDSCs, and (**C**) T-MDSCs, pre-treated or not with KCl, to inhibit the proliferation of T cells. MDSCs/T cell ratios are indicated in each figure. Statistical differences are shown by comparing the proliferation of T cells cultured in the absence of MDSCs with CD3^+^ T cells co-cultured with MDSCs pre-treated with or without 40 mM KCl, represented as K^+^. Comparisons were also performed between KCl pre-treated MDSCs with control MDSCs co-cultured with T cells. Control indicates MDSCs induced in complete media containing 5 mM KCl. The data are shown as the means ± SDs of at least three to four independent experiments. * *p* < 0.05, ** *p* < 0.01, *** *p* < 0.001, **** *p* < 0.0001 (one-way ANOVA with Tukey’s multiple comparisons test).

Our previous results showed that potassium abrogates the expression of Arg I, an enzyme that has been commonly used as a marker of MDSCs with immunosuppressive features. Therefore, we further assessed whether K^+^ ions alleviate the immunosuppressive function of MDSCs on T cell proliferation. Unexpectedly, despite the impaired expression of Arg I and iNOS by K^+^ ions, it did not influence the ability of MDSCs (cytokine-induced MDSCs, TES-MDSCs, and T-MDSCs) to suppress T cell proliferation (Figure 7A–C), suggesting that MDSCs exposed to K^+^ ions preserve a potent immunosuppressive function independent of Arg I and iNOS. Therefore, despite the high concentration of K^+^ ions in tumors affecting the expression of Arg I and iNOS by MDSCs, these cells adapt and maintain their pathological immunomodulatory functions in the TME, as widely recognized in the literature from human and mouse cancer studies.

## 4. Discussion

The TME is a unique niche governed by constant crosstalk within and across all intratumoral cellular compartments. The newly identified intratumoral ionic disturbance by K^+^ ions that affects immune cells has fueled our interest in understanding the effect of excess K^+^ ions on MDSCs. Toward this goal, we developed in vitro and ex vivo models to assess the functional, metabolic, and molecular responses of MDSCs to excess K^+^ ions. Here, we show that, although high concentration of K^+^ ions in cultures of MDSCs increased their mitochondrial respiration, limited their proliferation, and enhanced autophagy, in concert, intracellular signaling through pAMPK and CEBP/β was altered and reduced the expression of Arg I, but importantly, did not impair its immunosuppressive function. Therefore, MDSCs adapt to the hyperosmotic stress induced by excess K^+^ ions by performing metabolic and signaling rearrangements to sustain cellular survival via autophagy and maintain their immunosuppressive capacity independently of Arg I and iNOS.

MDSCs are immature myeloid immune cells that display an immunosuppressive phenotype in cancer [42,43] that is driven by molecular mechanisms, including signaling pathways induced by kinases AMPK, Akt, and ERK [28,29], and transcription factors such as C/EBP-β [31,32]. These intracellular signaling cascades result in metabolic changes [29,31,32], promoting autophagy [38,39], the production of mediators of immunomodulation, including Arg I, iNOS [25,43], ROS [44,45], and cytokines [43]. C/EBP-β is a critical transcription factor that modulates many biological processes in MDSCs, including the survival, differentiation, function, and expression of immunosuppressive molecules such as Arg I [31,32]. We found that C/EBP-β is downregulated by excess K^+^ ions and, given its critical role in cell growth [32], its reduced expression may explain the reduced ability of MDSCs to proliferate in the presence of elevated concentrations of KCl.

AMPKα has been shown to act as both a negative and positive regulator of MDSCs [29,46,47]. As a negative regulator, AMPK signaling was shown to have repressive effects on the expansion of MDSCs and their functions in inflamed tissues [29]; therefore, it is not surprising that reductions in pAMPK levels result from excess potassium, since this may be part of the adaptive strategies of MDSCs to sustain their functional phenotype. As a positive regulator, the active form of AMPK by phosphorylation has been correlated with an increased expression of Arg I and iNOS in MDSCs [47], suggesting that the downregulation of these two suppressive factors in potassium-treated MDSCs could be the result of the low activation of AMPK. AMPK and C/EBP-β play critical roles in regulating autophagy under different pathological states [39,40,48,49]. Furthermore, high concentrations of K^+^ ions upregulate the phosphorylation of Akt and ERK in MDSCs, a positive regulation previously shown in other cell types [36,37,50,51,52]. The reduced expression of C/EBP-β and levels of pAMPK may result in part from the increased expression of phosphorylated Akt, since previous reports have indicated that signaling by Akt suppresses C/EBPβ expression in myeloid cells [33] and negatively regulates AMPK activation [53]. Similarly, the activation of ERK inhibits the phosphorylation of AMPKα, as observed when the pharmacological inhibition of ERK restored AMPK signaling [35,54,55].

It has been shown that the interaction between autophagy and mitochondrial function supports the functional activity of MDSCs [29]. We have found that high levels of K^+^ ions increased mitochondrial respiration without affecting the glycolytic pathway or glucose uptake in MDSCs, suggesting that functional changes in MDSCs induced by high concentration of K^+^ ions are more likely associated with mitochondrial respiration independent of mechanisms related to energy deficits. Additionally, excess K^+^ ions increased the expression of the autophagy marker LC3β. These findings agree with a recent study showing that high K^+^ ion levels induce autophagy and mitochondrial respiration in T cells [2]. Therefore, our findings suggest that potassium enhances the capacity for on-demand oxygen consumption and induces autophagy, likely to sustain MDSCs functions. We also observed that the inhibition of autophagy with 3-MA restored the expression of Arg I and prevented the downregulation of C/EBP-β and levels of pAMPKα. The role of C/EBP-β [40] and AMPK [56] in regulating autophagy has been previously described. Therefore, our findings suggest a relationship between the signaling pathways of C/EBP-β and pAMPKα, autophagy, and the expression of Arg1 and iNOS; however, the exact mechanism remains to be elucidated.

One of the intriguing findings of our study is that excess K^+^ ions did not influence the immunosuppressive activity of MDSCs on T cell proliferation, despite the diminished expression of Arg I. However, this result agrees with a recent study showing that Arg I is not required for the MDSC-mediated inhibition of T cells [57]. The fact that KCl-treated MDSCs can inhibit T cell proliferation in the absence of Arg I and iNOS expression suggests that alternative mechanism(s), instead of depleting L-arginine, remain intact or are even enhanced to overcome the lack of Arg I and iNOS activity. MDSCs employ a diverse set of distinct mechanisms to suppress the proliferation and effector functions of T cells that were beyond the scope of this study. These mechanisms, which we did not explore in our study, include the secretion of soluble mediators such as reactive oxygen species (ROS) and cell contact-mediated events, including the expression of immune regulatory molecules such as PD-L1 and CTLA4 and the metabolic depletion of cystine, and by expressing ectoenzymes regulating adenosine production [44]. Ultimately, MDSCs are present within the TME in both human and mouse tumors, where they retain their immunosuppressive function despite the high intratumoral potassium concentration. This supports the idea that MDSCs adapt to hypertonic stress by upregulating alternative mechanisms to maintain their immunoregulatory properties.

MDSCs, like TAMs, play a pivotal role in the cellular network regulating immune responses in the TME through the enzymatic activity of Arg I, among other mechanisms [11,13,58]. A recent study has shown that a high concentration of extracellular K^+^ ions promotes the polarization of bone marrow-derived macrophages (BMDMs) toward pro-tumor M2-like TAMs with elevated expressions of Arg I, VEGF, IL-10, and OXPHOS [6]. Intriguingly, we found the opposite effect of K^+^ ions on protein and RNA expression of Arg I in MDSCs. The reason for this difference is unknown; however, considering the phenotypic variations in these cell types, it could be related to the expression of channels for K^+^ ions. One limitation of our current study is that we did not characterize the expression of potassium channels in our MDSCs. Potassium channels are transmembrane proteins that regulate different biological processes [59,60]. Unfortunately, limited information is available about the expression levels and types of ion channels in MDSCs [61,62]. The type and levels of K^+^ ions channels in mBM-MDSCs may differ from those expressed by cancer, TAMs, or T cells, raising the possibility of metabolic competition within the TME. The relevance of potassium channels is also evidenced in neutrophils, the fully differentiated counterparts of the G-MDSCs subset, which have a high permeability for potassium, mainly through ATP-sensitive potassium channels, impacting the neutrophil migration and plasma exudation of the inflammatory response [63], as well as the killing activity of these cells [64]. Importantly, potassium channels are highly regulated by characteristic settings of the TME milieu such as hypoxia, which not only inhibits potassium channels [65,66,67], but also modifies the function of calcium and voltage-activated potassium channels [68]. Therefore, although it was beyond the scope of this study, pre-clinical studies would help to identify the clinical implications of our findings in the K^+^ ion-enriched and hypoxic TME.

## 5. Conclusions

Our findings reveal that excess K^+^ ions promote autophagy, which impairs the expression of signaling molecules of MDSCs and their metabolism but does not affect the capacity of MDSCs to suppress the proliferation of T cells. Notably, our findings suggest that the MDSC-mediated immunosuppression of T cell proliferation is independent of the enzymatic activity of Arg I and iNOS when MDSCs are in the presence of a high concentration of K^+^ ions. The level of pAMPKα and the expression of C/EBP-β signaling pathways are involved in the adaptation of MDSCs to the hostile potassium-rich microenvironment but are not necessary for MDSCs’ immunosuppressive function. Identifying the molecular mechanisms involved in changes in MDSCs’ phenotype directed by K^+^ ions may open new avenues for therapies.

## Data Availability

The data are contained within the article and the Supplementary Materials.

## Supplementary Materials

The following supporting information can be downloaded at https://www.mdpi.com/article/10.3390/cells13201736/s1, Figure S1: Cytotoxic effect of excess K^+^ ions on cytokine-induced mBM-MDSCs; Figure S2: Gating strategy to identify MDSCs subsets from cytokine-induced mBM-MDSCs mediated by excess K^+^ ions; Figure S3: Excess K^+^ ions do not affect the viability of TES-induced MDSCs; Figure S4. Potassium ions increase autophagy as measured by LC3 protein expression in a time-dependent manner in mBM-MDSCs.
