# Supplementary material for: Excess Potassium Promotes Autophagy to Maintain the Immunosuppressive Capacity of Myeloid-Derived Suppressor Cells Independent of Arginase 1
Source: Cells. 2024 Oct 19;13(20):1736. doi: 10.3390/cells13201736 (PMC11505641; doi:10.3390/cells13201736)
Supplement: Supplementary file 1 [file cells-13-01736-s001.zip › cells-3142755-supplementary.pdf]

Supplementary Materials

Excess Potassium Promotes Autophagy to Maintain the Immunosuppressive Capacity of M**yeloid-Derived Suppressor Cells** Independent of Arginase 1

Ramesh Thylur Puttalingaiah [1,*], Matthew J. Dean [1], Liqin Zheng [1], Phaethon Philbrook [1,2], Dorota Wyczechowska [1], Timothy Kayes [1], Luis Del Valle [1], Denis Danos [1,3,4] and Maria Dulfary Sanchez-Pino [1,2,3,*]

[1] Stanley S. Scott Cancer Center, Louisiana State University Health Sciences Center, New Orleans, LA 70112, USA; mdean3@lsuhsc.edu (M.J.D.); lzheng@lsuhsc.edu (L.Z.); pphilb@lsuhsc.edu (P.P.); dwycze@lsuhsc.edu (D.W.); tkayes@lsuhsc.edu (T.K.); ldelva@lsuhsc.edu (L.D.V.); ddanos@lsuhsc.edu (D.D.)
[2] Department of Genetics, Louisiana State University Health Sciences Center, New Orleans, LA 70112, USA
[3] Department of Interdisciplinary Oncology, Louisiana State University Health Sciences Center, New Orleans, LA 70112, USA
[4] School of Public Health, Louisiana State University Health Sciences Center, New Orleans, LA 70112, USA
* Correspondence: msanc2@lsuhsc.edu (M.D.S.-P.); rthylu@lsuhsc.edu or rameshthylur@gmail.com (R.T.P.); Tel.: +1-504-210-2831 (M.D.S.-P.); +1-504-210-2046 (R.T.P.); Fax: +1-504-210-2970 (M.D.S.-P. & R.T.P.)

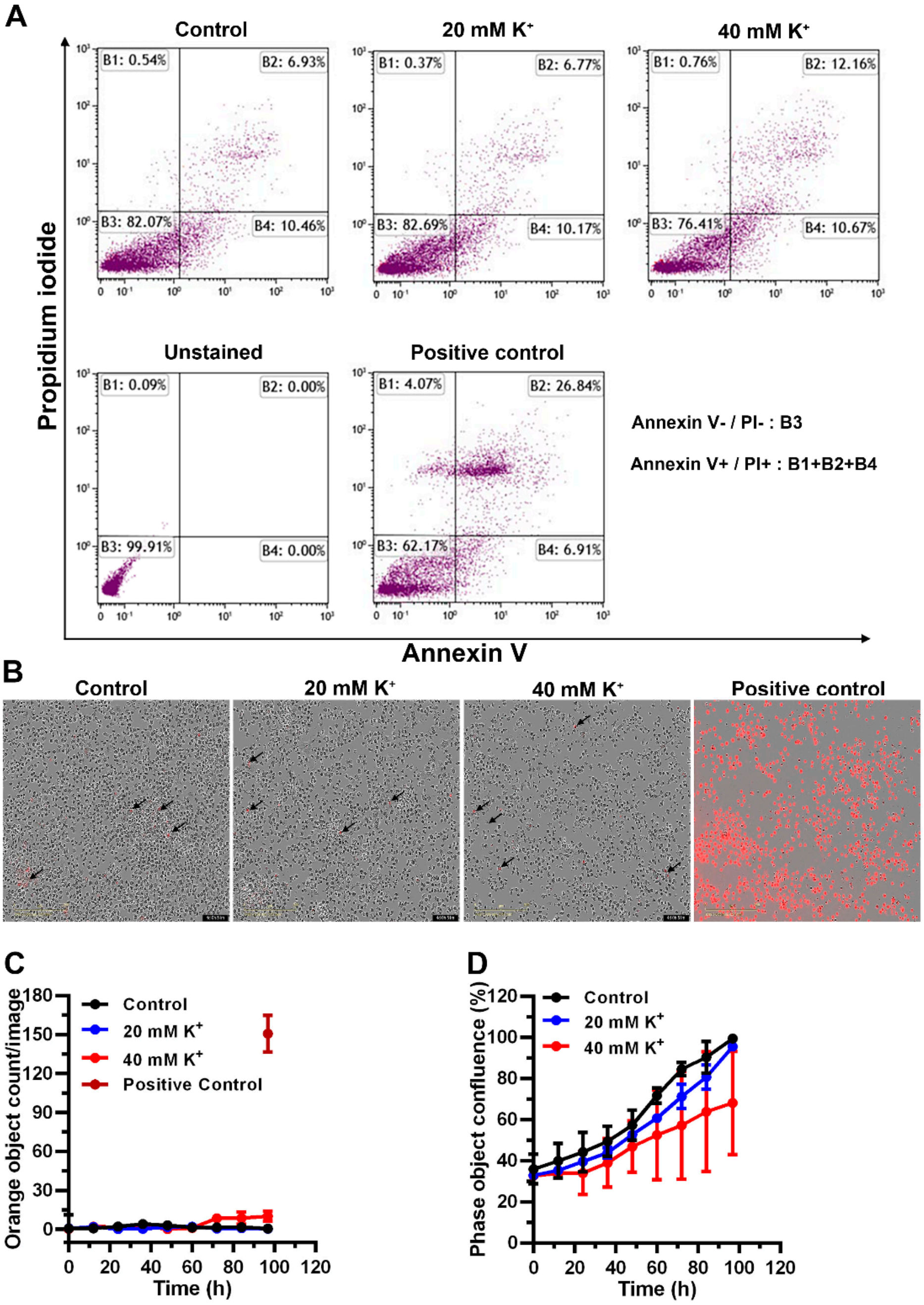

Figure S1. Cytotoxic effect of excess K^+ ions on cytokine-induced mBM-MDSCs.

(A) Representative dot plot illustrating the percentage of dead ($AnnV^+/PI^+$) and viable ($AnnV^-/PI^-$) mBM-MDSCs measured by the Annexin V/Propidium iodide (PI) staining method. Control indicates MDSCs induced in complete media that contains 5 mM KCl. A mixture of dead cells induced by exposure to high temperatures (3 minutes at 70^0 C) with alive cells was used as a

positive control for establishing instrument compensation and gating. Data represents one of three independent experiments. (B) Representative images of cells captured by the Incucyte Live-Cell Analysis System. Arrows indicate Incucyte Cytotox Red stained cells in control and treated with K^+. (C) Time-course for the levels of cell death as measured by Orange Object Count in mBM-MDSCs and the positive control that consisted of adding lysis solution into a well at the end of the cell culture. Data represents the mean ± standard deviation (SD) from 4 pictures per well in one experiment. (D) Time-dependent increase of cell confluence calculated by phase contrast images-based measurements.

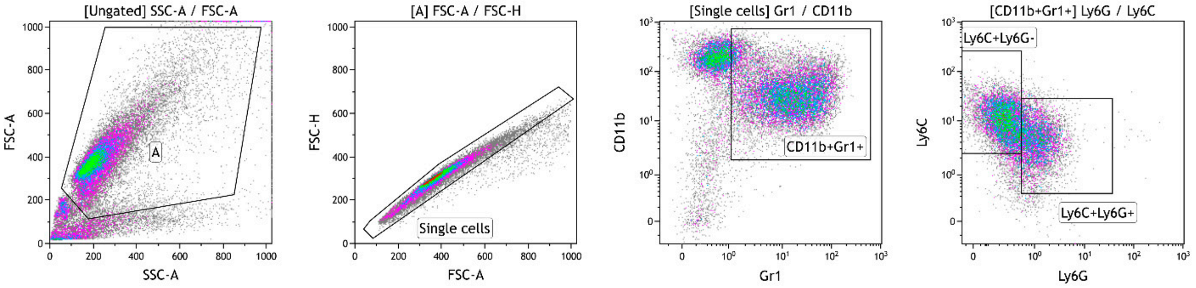

Figure S2. Gating strategy to identify MDSCs subsets from cytokine-induced mBM-MDSCs mediated by excess K^+ ions. Gating strategy to identify MDSCs subsets from cytokine-induced mBM-MDSCs after plotting FSC-A and FSC-H for doublet exclusion. MDSCs subsets were defined as a percent of $CD11b^+Gr1^+Ly6C^+Ly6G^-$ (Monocytic-MDSCs; M-GMDSCs) and percent of $CD11b^+Gr1^+Ly6C^{dim}Ly6G^+$ (Granulocytic-MDSCs; G-GMDSCs) by flow cytometry analysis after exposure to indicated concentrations of potassium chloride for 96 h (KCl; represented as K^+). Control indicates cytokine-induced MDSCs cultured in complete media that contains 5 mM KCl.

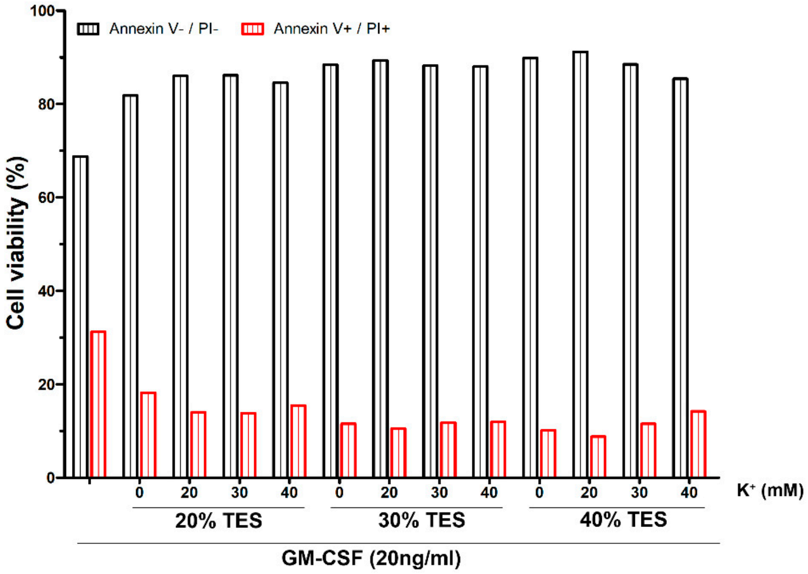

Figure S3. Excess K^+ ions do not affect the viability of TES-induced MDSCs. The percentage of dead or viable cells was measured by Annexin V/Propidium iodide (PI) staining method. TES-induced MDSCs were cultured with 20 ng/ml of GM-CSF, in the presence or absence of 20%, 30%, and 40% of MC38-derived TES and 0, 20, 30, or 40 mM KCl for 96 h. Cells that were cultured with TES and GM-CSF without KCl and cells that were cultured with GM-CSF alone are both considered as controls. Data represents one independent experiment.

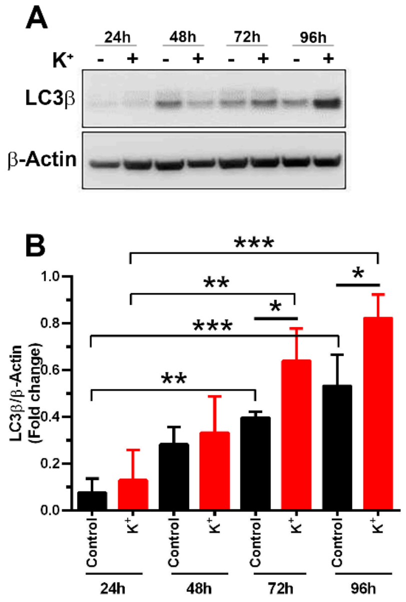

Figure S4. Potassium ions increase autophagy as measured by LC3 protein expression in a time-dependent manner in mBM-MDSCs. (A) Immunoblot and (B) densitometric quantification of LC3β in cytokine-induced MDSCs treated or not with KCl for 24h, 48h, 72h and 96h. Data represents the mean ± SD of three independent experiments. *P < 0.05, **P < 0.01, ***P < 0.001, (unpaired two-tailed *t*-test analysis or one-way ANOVA with Tukey's multiple comparisons test).
